# Bone marrow mesenchymal stromal cells metabolic reprogramming in systemic lupus erythematosus: remodeling of bone marrow microenvironment and regulation of immune cell fate

**DOI:** 10.3389/fimmu.2026.1725298

**Published:** 2026-02-27

**Authors:** Xiaoxiao Yang, Jiashuo Cheng, Jun Xiao, Zhifeng Gu, Chen Dong

**Affiliations:** Department of Rheumatology, Research Center of Clinical Medicine, Research Center of Clinical Immunology, Affiliated Hospital of Nantong University, Medical School of Nantong University, Nantong University, Nantong, China

**Keywords:** bone marrow mesenchymal stromal cells, bone marrow microenvironment, immune-mediated inflammatory disease, metabolic reprogramming, systemic lupus erythematosus

## Abstract

Systemic Lupus Erythematosus (SLE) is a chronic immune-mediated inflammatory disease characterized by dysregulated immune tolerance, abnormal secretion of autoantibodies, and multi-organ damage. Among them, mutations in genetic susceptibility genes, abnormal epigenetic modifications, excessive oxidative stress, abnormal accumulation of inflammatory factors, and intestinal flora disorders are all key specific factors that lead to immune dysfunction, abnormal production of autoantibodies, and multi-organ damage in patients with SLE. The bone marrow microenvironment, as a key niche for immune cell development, plays a pivotal role in the pathogenesis of SLE, especially through the metabolic reprogramming of bone marrow mesenchymal stromal cells (BMSCs). Recently, studies have demonstrated that under the influence of the bone marrow microenvironment, BMSCs can undergo metabolic reprogramming, regulated by the aforementioned abnormal factors related to SLE, the key metabolic pathways such as glucose metabolism, lipid metabolism and mitochondrial metabolism are disrupted, thereby affecting their regulatory functions on various immune cells. This process plays a role in the development and progression of immune-mediated inflammatory diseases like SLE. This article provides a comprehensive review of the current knowledge regarding the metabolic regulatory mechanism of the BMSCs on the immune cells in SLE and discusses recent advances in clinical translation.

## 1 Introduction

Systemic lupus erythematosus (SLE) is a chronic immune-mediated inflammatory disease characterized by an imbalance in immune tolerance, abnormal autoantibody secretion and multi-organ damage. Its pathogenesis involves multiple factors, including the abnormal activation of innate and adaptive immunity, as well as impaired functions of apoptotic clearance (1, 2). Abnormalities in B-cell numbers and function play a crucial role in the development of lupus. These cells can cause tissue damage directly by producing pathogenic autoantibodies, or indirectly by acting as antigen-presenting cells and exacerbating malignant inflammatory cycles through the dysregulation of T cells and other immune cells, thereby accelerating disease progression. Recent studies have reported that lupus B cells exhibit abnormal characteristics during the early stages of development in the bone marrow. These include impaired early B cell development (3), disrupted CXCL12/CXCR4 signaling (4), and abnormal metabolic reprogramming (5). These findings suggest that, in SLE, the bone marrow microenvironment may influence the growth and development of B cells and other immune cells, as well as their characteristics and functions in the systemic circulation.

As key stromal cells within the bone marrow microenvironment, mesenchymal stromal cells (MSCs) have been shown to possess significant immunoregulatory functions and to play a crucial role in the pathogenesis and therapeutic strategies of various immune-mediated inflammatory diseases, including SLE. Recent studies have indicated that MSCs undergo metabolic reprogramming under the influence of the bone marrow microenvironment, which in turn alters their regulatory functions on various immune cells, including B cells (6–8). This, in turn, has been shown to contribute to the onset and progression of immune-mediated inflammatory diseases like lupus. The metabolic state of MSCs exerts a substantial influence on their immunoregulatory capacity. Under normal physiological conditions, MSCs primarily maintain energy homeostasis through glycolysis and oxidative phosphorylation. However, within inflammatory microenvironments, their metabolic pathways undergo significant reprogramming, enhancing their immunoregulatory capabilities. A comprehensive investigation into the regulatory mechanisms that underpin metabolic reprogramming of bone marrow MSCs in lupus, with particular emphasis on their effects on B cells and other immune cells, is of significant research value. Future efforts to identify key molecules and signaling pathways involved in MSC metabolic reprogramming may lead to the development of standardized, personalized, and precise clinical diagnostic and therapeutic strategies, thereby optimizing clinical outcomes for SLE patients. Consequently, the targeting of MSC metabolic reprogramming offers a novel therapeutic strategy for the treatment.

**Figure 1:**
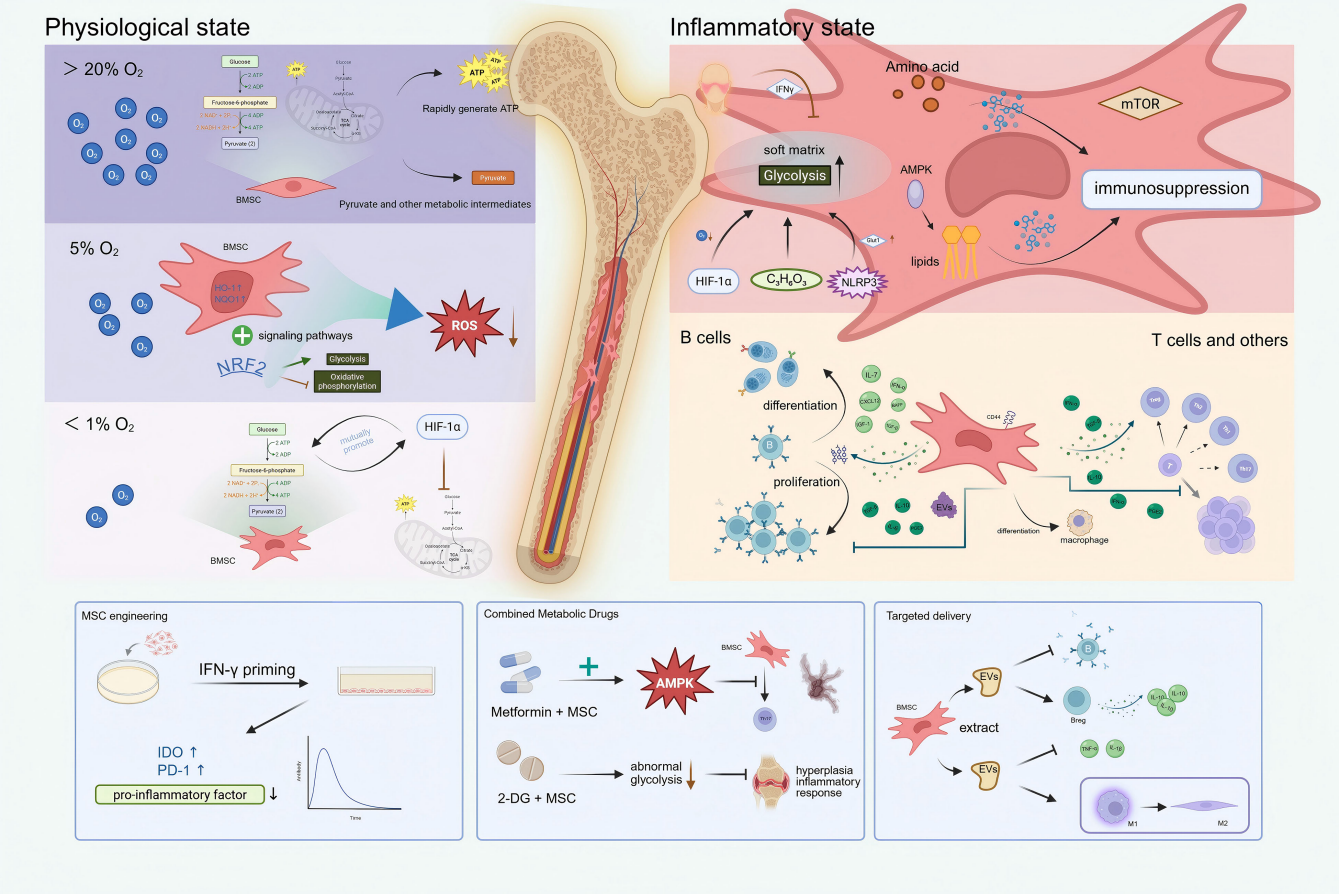
Overview of BMSC metabolic reprogramming.

This review will summarize the core mechanisms of BMSC metabolic reprogramming, dissect its regulatory networks governing B cells and other immune cells in systemic lupus erythematosus, and further outline clinical translation progress and future directions (**Figure 1**).

## 2 The core mechanism of metabolic reprogramming in BMSCs

### 2.1 Basal metabolic phenotype

Cell metabolism refers to all the chemical reactions that sustain cellular life activities, including anabolism and catabolism. Under normal conditions, MSCs can perform metabolic activities such as aerobic glycolysis, oxidative phosphorylation and fatty acid oxidation. Undifferentiated mesenchymal stromal cells primarily rely on glycolysis for energy acquisition to promote proliferation and self-renewal. Notably, this basic metabolic feature of MSCs is closely linked to systemic lupus erythematosus (SLE), an immune-mediated inflammatory disease characterized by dysregulated immune responses and widespread tissue injury. In SLE patients, the intrinsic metabolic homeostasis and plasticity of MSCs are significantly disrupted, which impairs their immune-regulatory functions (e.g., inhibiting abnormal T/B cell activation) and further accelerates disease progression. Certain metabolites can directly regulate the migratory capacity of mesenchymal matrix cells. In SLE, the expression and activity of TGM2 in MSCs are frequently downregulated; reduced TGM2-mediated Wnt/β-catenin pathway activation leads to impaired MSC migration and differentiation, weakening their ability to homing to SLE-involved lesion sites (e.g., kidneys, skin) and exert local immune regulation (9–12). Mitochondrial-derived reactive oxygen species (ROS) also play a pivotal role in migration. Importantly, SLE is accompanied by chronic excessive oxidative stress, and MSCs isolated from SLE patients exhibit abnormally elevated ROS levels, which not only inhibit their migration and proliferation but also induce premature senescence of MSCs, further reducing their therapeutic potential in SLE treatment (13, 14).

The oxygen environment is a key regulator of the metabolic status of MSCs. Under sufficient oxygen conditions, mesenchymal stromal cells exhibit relatively high glycolytic activity, a phenomenon analogous to the ‘Warburg effect’ observed in cancer cells. In SLE, the inflammatory microenvironment (characterized by abnormal oxygen tension, excessive pro-inflammatory cytokines such as IFN-γ and TNF-α) disrupts the normal oxygen-dependent metabolic balance of MSCs. SLE-derived MSCs often show dysregulated glycolytic activity even under sufficient oxygen conditions, with reduced oxidative phosphorylation capacity, which impairs their energy supply and further compromises their immune-regulatory function (15, 16). Under moderate hypoxic conditions (e.g. 5% O_2_), MSCs exhibit high proliferation and metabolism, relying on both oxidative phosphorylation and glycolysis pathways simultaneously (17). However, in SLE, the hypoxic adaptation ability of MSCs is significantly weakened; even under moderate hypoxic conditions, SLE-derived MSCs fail to effectively reduce ROS levels or enhance antioxidant capacity, leading to persistent oxidative damage and impaired functional integrity (18). When exposed to oxidative stress, MSCs mitigate damage by activating the nuclear factor E2-related factor 2 (NRF2) signaling pathway. In SLE patients, the NRF2 signaling pathway in MSCs is often downregulated, resulting in insufficient upregulation of antioxidant genes (HO-1, NQO1) and impaired ability to maintain intracellular redox balance under oxidative stress; this further aggravates MSC dysfunction and promotes the progression of autoimmune responses in SLE (19, 20). Chronic oxidative stress exhibits bidirectional regulation. In SLE, chronic excessive oxidative stress (instead of the normal bidirectional regulation) continuously impairs MSC function, and the HIF-1α-mediated metabolic reprogramming under deep hypoxia is disrupted. This disruption leads to failure in maintaining MSC quiescence and abnormal activation of glycolytic metabolism, further disturbing the immune-regulatory balance mediated by MSCs and exacerbating SLE pathogenesis (21, 22). However, under deep hypoxia (<1% O_2_), MSC metabolism primarily relies on anaerobic glycolysis rather than oxidative phosphorylation (21). This metabolic reprogramming helps to maintain the quiescence of MSCs. In this context, hypoxia activates hypoxia-inducible factor (HIF-1α) (22), thereby promoting the expression of glycolytic enzymes while suppressing mitochondrial oxidative phosphorylation.

All of the above demonstrate the high metabolic plasticity of MSCs under basal conditions, which is significantly disrupted in SLE. They can dynamically adjust the ratio of glycolysis to oxidative phosphorylation. In SLE, MSCs lose this ability to dynamically adjust the glycolysis-oxidative phosphorylation balance in response to environmental and cellular changes; this inability to adapt to the abnormal SLE microenvironment (excessive oxidative stress, abnormal oxygen tension) impairs their self-renewal, migration, and immune-regulatory capabilities, and further contributes to the pathogenesis and progression of SLE. This enables them to adapt to fluctuations in nutrients and oxygen levels within the stem cell microenvironment, while also providing the basis for self-renewal and subsequent differentiation.

Furthermore, the pathological features of SLE patients, including persistent inflammation, enhanced oxidative stress, and local microenvironmental hypoxia, can precisely induce abnormal metabolic reprogramming in BMSCs. This reprogramming not only disrupts their normal metabolic balance but also directly impairs their regulatory functions on immune cells such as T cells, B cells, and peripheral blood mononuclear cells (PBMCs). Studies have confirmed that SLE-associated metabolic abnormalities in BMSCs promote the upregulation of pro-inflammatory factors such as IL-6 and IFN-β, activate aberrant signaling pathways including IL-6/STAT3 and MAVS-IFN-β, induce an imbalance in Th17/Treg immune cell subsets, and simultaneously trigger phenotypic changes such as premature senescence, increased apoptosis, and functional impairment. These alterations collectively contribute to the development of immune dysregulation and disease progression in SLE, representing a core mechanism underlying BMSC dysfunction in SLE pathogenesis.

## 3 Metabolic reprogramming in the inflammatory microenvironment

Cellular metabolic reprogramming is the process by which cells adapt to new functional demands or survival environments under specific physiological or pathological conditions, involving alterations to metabolic pathways and metabolic product production. As a crucial mechanism through which cells respond to environmental changes, maintain survival and execute specific functions, metabolic reprogramming involves multidimensional changes, including regulation of gene expression, activation of signaling pathways, epigenetic modifications, and alterations in metabolites (23–25). This process is usually associated with changes in the expression of important metabolic enzymes (e.g. hexokinase) and transcription factors (e.g. c-Myc, HIF-1α) (26). In the chronic inflammatory pathological microenvironment of immune-mediated inflammatory diseases such as SLE, the glucose metabolism of bone marrow mesenchymal matrix cells (BMSCs) undergoes significant abnormal reprogramming, and this process is deeply associated with the immune disorder and disease progression of SLE.

In inflammatory or pathological microenvironments, the glucose metabolism of BMSCs undergoes significant reprogramming. Inflammatory cytokines are key triggers in this process. For example, IFN-γ pretreatment shifts BMSCs towards aerobic glycolysis, thereby enhancing their T cell-suppressive effects (6). Under oxidative stress, BMSCs increase glycolytic flux while reducing mitochondrial respiration. At the same time, they enhance NADPH synthesis via the pentose phosphate pathway to maintain cellular redox balance (19, 20). In the inflammatory environment of SLE, the high expression of proinflammatory factors such as IFN-γ further induces metabolic reprogramming in BMSCs, characterized by reduced glycolysis and increased oxidative phosphorylation (27). In patients with SLE, pro-inflammatory factors such as IFN-γ and IL-6 are persistently highly expressed, further driving the characteristic metabolic reprogramming of BMSCs - manifested as a decrease in glycolysis levels and an enhancement in oxidative phosphorylation. This abnormal pattern disrupts the normal immune regulatory function of BMSCs and instead exacerbates immune disorders. Lactic acid, a key product of glucose metabolism, plays a crucial role in regulating BMSC function. As the primary end product of glycolysis, lactic acid accumulation alters redox states within BMSCs (e.g. the NAD+/NADH ratio), thereby affecting the balance of cellular energy metabolism and modulating their immunoregulatory capacity (6, 28). Studies reveal that low concentrations of sodium lactate (1 mM) can activate the KDM6B demethylase, thereby upregulating glycolysis-related metabolic processes and altering the metabolic profile of BMSCs. Ultimately, this enhances their stemness characteristics (e.g. self-renewal capacity) (29, 30). In the pathological microenvironment of SLE, the lactate metabolism is imbalanced, which may further exacerbate the metabolic abnormalities of BMSCs and aggravate their functional impairment. The glucose metabolism of BMSCs is regulated by multiple signaling pathways and molecules. Upon activation in hypoxic conditions, HIF-1α promotes the expression of glycolytic enzymes, driving a metabolic shift towards glycolysis. Concurrently, lactate can influence BMSC proliferation and differentiation by modulating the HIF-1α signaling pathway (22, 29). The NLRP3 inflammasome influences BMSC glucose metabolism by regulating glucose transporter 1 (Glut1)-mediated energy metabolism reprogramming (31). Furthermore, the mechanical properties of the extracellular matrix are involved in this regulation; softer matrices enhance glycolysis in BMSCs through mechanical stress (32). In the pathological microenvironment of SLE, HIF-1α and NLRP3 inflammasomes are all in an abnormally activated state, jointly mediating the glucose metabolism disorder of BMSCs and becoming an important regulatory node for the functional abnormalities of BMSCs in SLE.

The lipid metabolism of BMSCs forms a synergistic regulatory network through core pathways such as adenosine monophosphate-activated protein kinase (AMPK) during cellular differentiation, immunoregulation, and stress adaptation. The regulation of mitochondrial function and energy metabolism is crucial during adipogenic differentiation; studies indicate that AMPK influences the adipogenic differentiation of MSCs by modulating mitochondrial function and energy metabolism (33). Lipid metabolites also modulate the immunoregulatory functions of MSCs, thereby enhancing their immunosuppressive capacity. Regulation of lipid metabolism is equally vital for the survival of MSCs under stress-induced conditions. For example, under hypoxic conditions, MSCs maintain the supply of cellular energy and antioxidant capacity by regulating lipid metabolism (33). Previous studies have indicated that BMSCs in SLE patients exhibit abnormal fat metabolism, which may disrupt the AMPK pathway and affect mitochondrial function, further weakening their immune regulatory ability and contributing to the progression of the disease.

Amino acid metabolism also plays a crucial role in the energy metabolism of MSCs. Amino acids such as glutamine are primary substrates for mitochondrial respiration and the tricarboxylic acid cycle. They provide cells with energy and metabolic intermediates and influence MSC differentiation by regulating intracellular signaling pathways such as the mTOR pathway. For example, glutamine metabolism regulates the osteogenic and adipogenic differentiation of MSCs via the mTOR pathway (34). Furthermore, amino acid metabolites enhance the immunomodulatory capacity of MSCs by modulating immune cell activity (6, 35, 36). *In vitro*, MSCs induce the production of indoleamine 2,3-dioxygenase (IDO) to catalyze the conversion of tryptophan (Trp) into kynurenine (Kyn). This inhibits the proliferation of effector T cells and induces the expansion of regulatory T cells (Tregs), demonstrating therapeutic efficacy against graft-versus-host disease (GVHD) (37, 38). When interacting with macrophages, bone marrow-derived MSCs can upregulate arginase 1 (Arg1) to deplete arginine in the microenvironment. This inhibits nitric oxide (NO) synthesis and suppresses macrophage M1 polarization (39, 40). In SLE patients, the expressions of IDO and Arg1 in BMSCs are abnormally downregulated, which leads to a weakened ability of these cells to regulate immune cells through amino acid metabolism. As a result, they are unable to effectively correct the imbalance of T cell and macrophage subpopulations, further exacerbating the immune disorder in SLE. (**Table 1**).

**Table 1:** Metabolic characteristics of BMSCs in SLE.

Research model	Core metabolism	Core metabolic changes
SLE patients' BMSC	Glycolysis	Decreased
	OXPHOS	Increased
	Mitochondrial function	Remodeled, with increased ROS and mtDNA levels
	Succinate and lactate metabolism	Increased secretion
	GP91 protein expression	Upregulated
	IFN-α-G-CSF signaling pathway	Activated
MRL/lpr mouse BMSC	Glucose uptake	Increased
	Reactive oxygen species (ROS) production	Increased
	OXPHOS pathway	Increased expression of related genes and enhanced activity
	Mitochondrial respiratory chain function	Increased enzyme activity and ATP synthesis efficiency
NZM2410 mouse BMSC	OXPHOS-dependent metabolic remodeling	Reversible

1. BMSC, Bone Marrow Mesenchymal Stem Cells; 2. OXPHOS, Oxidative Phosphorylation; 3. ROS, Reactive Oxygen Species; 4. mtDNA, Mitochondrial DNA; 5. GPR91, G protein-coupled receptor 91; 6. IFN-α, Interferon-α; 7. G-CSF, Granulocyte Colony-Stimulating Factor; 8. ATP, Adenosine Triphosphate; 9. LDG, Low-Density Granulocytes.

## 4 The key initiating signals that drive the metabolic reprogramming of BMSCs in the bone marrow microenvironment of systemic lupus erythematosus

In the bone marrow microenvironment of SLE, there are multiple characteristic key initiating signals that jointly drive the specific metabolic reprogramming of BMSCs, forming a unique pathological metabolic regulatory network. Among them, high levels of type I interferons (especially IFN-α) are one of the core initiating signals. As a hallmark pathological factor of SLE, it not only directly induces the metabolic spectrum of BMSCs to shift towards enhanced oxidative phosphorylation and weakened glycolysis, but also indirectly affects the metabolic interaction between BMSCs and immune cells by upregulating the secretion of survival-promoting molecules such as BAFF. The deposition of circulating immune complexes (such as anti-dsDNA antibodies) in the bone marrow microenvironment is also an important triggering factor. It can activate intracellular inflammatory signaling pathways (such as the NF-κB pathway), disrupt the basic metabolic balance of BMSCs, promote their metabolic reprogramming, and enhance immune regulatory activity. In addition, the unique inflammatory cytokine profile in the SLE bone marrow microenvironment (including IFN-γ, TNF-α, IL-6, etc.) forms a synergistic effect, further amplifying the metabolic reprogramming signals: IFN-γ dominates the adjustment of the ratio of glycolysis and oxidative phosphorylation, TNF-α strengthens the regulation of mitochondrial function changes and ROS generation, and IL-6 participates in the coordinated regulation of amino acid metabolism and fat metabolism pathways, jointly shaping the metabolic phenotype of BMSCs that is adapted to the SLE pathological environment, laying a metabolic foundation for their subsequent regulation of B cell function.

## 5 The regulation of B cells by BMSC metabolic reprogramming in SLE

MSCs primarily regulate B cells at two levels: development and activation. This occurs through various mechanisms within the bone marrow microenvironment, such as cytokines, chemokines, metabolic signals, and direct cell-to-cell interactions. During B cell development, MSCs and their microenvironment influence B cell differentiation, maturation, and homing via various signaling pathways. IL-7, which is secreted by osteoblasts, is a key factor in the early maturation of B cells. However, its dysregulation in combination with IFN-α can drive B cell differentiation towards autoantibody-producing plasma cells (3, 41, 42). Excessive production of the chemokine CXCL12 by osteoblasts enhances CXCR4^+^ B cell homing to the bone marrow via the CXCL12/CXCR4 signaling pathway. This leads to abnormal accumulation of B cells in the marrow and reduced output to the periphery, a phenomenon observed in patients with active SLE (3, 4). Insulin-like growth factor 1 (IGF-1), which is secreted by COL2.3^+^ stromal cells, modulates B-cell metabolic pathways. Abnormal secretion of IGF-1 may trigger metabolic hyperactivity in autoreactive B cells (43). Furthermore, transforming growth factor beta (TGF-β), which is secreted by MSCs, may disrupt the epigenetic regulation of B-cell tolerance (44). In severe MRL/lpr lupus mouse models, bone marrow MSC transplantation modulates the differentiation bias of myeloid cells in hematopoietic stem and progenitor cells, thereby indirectly affecting B-cell development (45).

At the level of B-cell activation, MSCs suppress abnormal activation and modulate function by secreting soluble factors, undergoing metabolic reprogramming, and engaging in intercellular interactions. MSCs secrete soluble factors such as TGF-β, IL-10, and prostaglandin E2 (PGE2), or they inhibit B-cell activation, proliferation and antibody secretion via direct cell-to-cell contact. They also increase the proportion of memory B cells, thereby modulating humoral immune responses (46–49) a phenomenon that has also been observed in severe MRL/lpr lupus mice (45). In SLE, MSCs suppress B-cell activation and autoantibody production, and metabolic reprogramming enhances this effect further. MSCs also promote the differentiation of IL-10-secreting regulatory B cells to alleviate inflammation (50, 51). In other immune-mediated inflammatory diseases such as rheumatoid arthritis (RA), metabolically reprogrammed MSCs modulate T/B cell metabolic pathways to suppress abnormal activation, thereby reducing proinflammatory factors and autoantibody production (52). MSC-derived extracellular vesicles also regulate B-cell responses by transferring microRNAs (miRNAs) and interleukin-6 (IL-6) (53, 54). Conversely, the abnormal secretion of BAFF by MSCs and other stromal cells in the bone marrow microenvironment promotes the survival of autoreactive B cells via anti-apoptotic pathways. High IFN-α expression further increases BAFF secretion and enhances B cell responsiveness to BAFF, creating a pro-survival environment for autoreactive B cells (55–58).

## 6 The regulation of T cells by BMSC metabolic reprogramming in SLE

MSCs regulate T cells via two key pathways: development and activation. This is achieved via multiple mechanisms, including the secretion of soluble factors, direct cell-to-cell contact, and metabolic intervention. At the level of T cell development, bone marrow-derived MSCs can indirectly influence the balance of T cell development and differentiation. TGF-β, which is secreted by MSCs, stimulates the immunosuppressive activity of Tregs, thereby providing an immunoregulatory microenvironment for T cell differentiation (44). Macrophages generated from MSCs reduce the formation of Th1 and Th17 lymphocytes, promoting the generation of Tregs (39). Furthermore, cytokines regulated by cells such as MSCs in the bone marrow microenvironment (e.g. IFN-α) can influence early T cell differentiation by activating the JAK-STAT pathway. Meanwhile, metabolic reprogramming of MSCs may maintain normal T cell development by correcting such signaling dysregulation (57, 59). In the inflammatory environment of patients with SLE, proinflammatory factors such as IFN-γ are highly expressed (60, 61). This environment can induce metabolic reprogramming in MSCs: glycolysis is reduced, oxidative phosphorylation is enhanced, and the expression of indoleamine IDO and programmed death ligand 1 (PD-L1) is simultaneously increased (27). This altered metabolic state significantly enhances the ability of MSCs to suppress T cell proliferation, thereby exerting more effective immunoregulatory effects.

At the level of T cell activation, MSCs suppress abnormal activation and regulate functional balance through multiple mechanisms. MSCs secrete soluble factors, such as TGF-β, IL-10 and PGE2, which directly inhibit T cell proliferation and activity, and reduce the secretion of proinflammatory factors, such as IFN-γ and TNF-α, by Th1 cells (46, 47). MSCs also inhibit Th1 cell activation and proliferation through direct cell-to-cell contact, while promoting Th2 cell differentiation (62). This corrects the Th1/Th2 imbalance and attenuates inflammatory responses (63). In inflammatory environments, activated MSCs release increased levels of IL-10 and TGF-β, which further enhances the suppression of T cell hyperactivation (64). Conversely, MSCs induce Treg expansion via direct contact through surface molecules (e.g. HLA-G and CD44), as well as indirectly suppressing T cell function by depleting the microenvironment necessary for T cell activation through metabolic pathways (e.g. IDO-mediated tryptophan degradation and lactate accumulation) (47). In terms of addressing T cell subset dysregulation, MSC-secreted IL-10 and TGF-β suppress Th17 cell differentiation while promoting Treg generation. This corrects the Th17/Treg imbalance (64, 65). Metabolically reprogrammed MSCs (e.g. those pretreated with IFN-γ) are more effective at suppressing the proliferation of proinflammatory T cells, such as Th17, while enhancing Treg generation. This alleviates immune dysregulation in diseases such as SLE (27, 66).

## 7 Regulation of other immune cells by BMSC metabolic reprogramming in SLE

Under normal conditions, BMSCs can effectively suppress excessive inflammatory responses by releasing immunomodulatory factors such as PGE2 and IDO (67). However, in the SLE environment, B cell-derived immune complexes stimulate mesenchymal matrix cells and endothelial cells to secrete IL-6 (68, 69). Not only does IL-6 promote B cell differentiation into plasma cells and enhance autoantibody production, it also indirectly exacerbates neutrophil extracellular trap (NET) formation (NETosis) by inducing anti-citrullinated protein antibodies (ACPA) (69). Furthermore, inflammatory signals in the bone marrow microenvironment, such as granulocyte colony-stimulating factor (G-CSF) secreted by endothelial and stromal cells, can be activated by cytokines such as TNF-α derived from B cells (56, 70). This abnormal signaling activation shifts hematopoietic differentiation from lymphopoiesis towards granulopoiesis, leading to an abnormal increase in neutrophil numbers, including low-density granulocytes (LDGs)—a cell subset with significant pathogenicity in SLE (71, 72). LDGs originate from abnormal myeloid differentiation processes in the bone marrow (73), and their numbers correlate closely with type I IFN-α levels in the bone marrow (74). This population exhibits characteristics of pathogenesis, such as spontaneous NET formation and constitutive overexpression of ROS (71, 72). In pristane-induced lupus mouse models, both bone marrow LDG counts and NET release capacity were significantly higher than in controls, confirming the bone marrow microenvironment as a major source of pathogenic neutrophils in SLE (75). However, the precise association between LDGs and disease activity in human SLE requires validation through larger clinical cohorts (74). It is worth noting that the metabolic reprogramming of BMSCs (especially the transition to oxidative phosphorylation (OXPHOS)) may provide key metabolic substrates or microenvironmental signals for the aforementioned abnormal myeloid differentiation and LDG generation by reshaping the energy production pattern and the secretion spectrum of metabolic intermediates. Under normal physiological conditions, BMSCs mainly rely on glycolysis for energy metabolism, with rapid but low efficiency energy production and the secretion of a small amount of metabolic products such as lactic acid (76, 77); when metabolic reprogramming occurs towards OXPHOS under inflammatory microenvironment induction, the energy production efficiency of BMSCs significantly increases, and the aerobic metabolic process dependent on mitochondria can produce more ATP to maintain their survival and immune regulatory functions in the inflammatory environment, accompanied by a significant change in the secretion spectrum of metabolic intermediates (78). Among them, succinate, as a key intermediate product of the tricarboxylic acid cycle, may accumulate intracellularly and be secreted when OXPHOS is enhanced, and succinate not only can regulate the function of inflammatory cells by activating the GPR91 receptor, but also may act as a signaling molecule to regulate the differentiation direction of bone marrow hematopoietic stem cells and promote the bias towards granulocyte generation in myeloid differentiation, thereby providing metabolic driving signals for the abnormal proliferation of LDG. In addition, although OXPHOS is the main metabolic mode, BMSCs may still retain a certain level of glycolysis under inflammatory conditions, resulting in changes in lactate secretion, and lactate, as an important metabolic regulator in the immune microenvironment, can further amplify the abnormal induction effect of the inflammatory microenvironment on myeloid differentiation by regulating the signaling pathways of bone marrow stromal cells and hematopoietic cells, promoting the generation of LDG and the acquisition of pathogenic phenotypes. Currently, the specific secretion levels of metabolic intermediates such as succinate and lactic acid after BMSCs undergo metabolic reprogramming towards OXPHOS, as well as their synergistic mechanism with inflammatory signals such as type I IFN-α and G-CSF in the bone marrow microenvironment, still require more *in vitro* cell experiments and *in vivo* animal model studies to clarify, which also provides a potential direction for targeting the metabolic reprogramming of BMSCs to intervene in the pathological process mediated by LDG in SLE.

Metabolically reprogrammed MSCs can suppress M1 macrophage polarization and induce the anti-inflammatory M2 phenotype by secreting specific metabolites or cytokines. This metabolic regulation-based immune regulatory mechanism also has potential associations with various immune-mediated inflammatory diseases. For instance, in multiple sclerosis, the lipid metabolism disorder of MSCs may affect their regulation of immune cells in the central nervous system; in inflammatory bowel disease, the fatty acid oxidation disorder and glycolytic conversion of local MSCs in the intestine may contribute to the imbalance of intestinal barrier immunity; and in systemic lupus erythematosus, the mitochondrial dysfunction and abnormal metabolic reprogramming of MSCs may weaken their regulatory ability on autoreactive B cells and dendritic cells, thereby affecting the disease progression. In RA, enhanced glycolysis in synovial macrophages leads to succinate accumulation, activating the GPR91 receptor. This process further increases glycolysis-related gene expression and the release of inflammatory mediators (79). MSCs suppress synovial inflammation and modulate metabolites and signaling pathways in the inflammatory microenvironment through metabolic reprogramming. This inhibits pro-inflammatory cell activation and cytokine release (80, 81). This influences the polarization state of macrophages, suppressing pro-inflammatory M1 polarization while inducing anti-inflammatory M2 polarization, thereby alleviating inflammatory responses in RA (82). Furthermore, exosomes derived from bone marrow mesenchymal matrix cells can promote M1-to-M2 conversion by interfering with the secretion of proinflammatory cytokines by macrophages, thereby reducing the expression of proinflammatory cytokines (TNF-α, IL-1β, IL-6, and IL-8) and increasing the expression of the anti-inflammatory cytokine IL-10 (83). In lipopolysaccharide-treated alveolar macrophages, MSC-derived exosomes inhibit M1 polarization and promote M2 polarization by suppressing cellular glycolysis through the downregulation of hypoxia-inducible factor 1α (84). Further evidence is required to demonstrate the anti-inflammatory effects of MSCs on macrophages in other inflammatory and degenerative diseases, such as systemic lupus erythematosus.

MSCs can also influence the maturation and function of dendritic cells (DCs), rendering them more effective at inducing Treg generation and thereby indirectly regulating the Th17/Treg balance (85). Specifically: MSCs can suppress the differentiation of monocytes into DCs and reduce the maturation of already-differentiated DCs (86, 87). This is manifested by the downregulation of co-stimulatory molecule expression (e.g. CD80 and CD86) on DC surfaces, which weakens their ability to activate T cells. Simultaneously, they suppress DC secretion of proinflammatory cytokines (e.g. IL-12) while promoting anti-inflammatory factors (e.g. IL-10) to modulate immune response characteristics. Co-culturing MSCs with DCs induces the generation of immunoregulatory tolerogenic dendritic cells (tol-DCs), which can induce T cell immune tolerance. For instance, MSC-induced tolerogenic dendritic cells (tol-DCs) have been demonstrated to enhance graft survival in organ transplantation (88). Furthermore, MSCs can reduce the antigen-presenting capacity of DCs, thereby diminishing T cell activation (89). In SLE, MSCs promote the proliferation of tolerant CD1c^+^ DCs in the peripheral blood of patients and inhibit their apoptosis, thereby maintaining immune tolerance and preventing the immune system from attacking its own tissues (90). Moreover, MSCs induce antigen-specific tolerance by enhancing the inactivation of autoreactive cells through the following mechanisms: suppressing the maturation of antigen-presenting DCs; blocking T cell receptor (TCR) pathways; secreting inhibitory molecules; increasing apoptotic activity to eliminate the cells; and activating Tregs to enhance their proliferation and induce tolerogenic DCs (91) (**Table 2**).

**Table 2:** The core characteristics of metabolic reprogramming and immune regulation of MSC (mesenchymal stem cells) in systemic lupus erythematosus.

MSC source/type	Regulated target	Molecules/metabolic pathways	Change	Metabolic characteristics	Final effect
Bone marrow BMSC from lupus mice (MRL/lpr)	Intracellular metabolism	IL-6, p-STAT3, p62, Beclin1/STAT3	up	Abnormal intracellular metabolism, decreased autophagy	MSC premature senescence/impaired function, accelerated SLE progression
		Autophagy	down		
Bone marrow BMSC from lupus mice (MRL/lpr)	T lymphocytes	Akt, YAP, IL-1β, IL-6/TNF-α, Rorc γt	up	Enhanced glycolysis	Th17/Treg imbalance, weakened inhibition, aggravated lupus
		Foxp3	down		
Bone marrow BMSC from lupus mice (BWFI)	Systemic lipid metabolism	mTOR, RPS6K, Akt, IBS-1	up	Dysregulated blood lipids and hormone metabolism	Aggravated systemic metabolic disorders, accelerated renal injury
Bone marrow BMSC from SLE patients	Cell cycle	p21, p27, p53/Akt	up	Abnormal cell cycle metabolism, activated mitochondrial pathway	Cell cycle arrest at G0/G1 phase, decreased proliferation, increased apoptosis
		CDK2/CDK4	down		
Bone marrow BMSC from SLE patients	Oxidative stress	IFN-β, MAVS, ROS, p53/p16, IFN-β-TNF-α positive feedback	up	Abnormal oxidative stress metabolism, enhanced DNA damage repair	MSC premature senescence, increased pro-inflammatory factors, aggravated immune disorders
Bone marrow BMSC from SLE patients	Peripheral blood mononuclear cells (PBMC)	MEK, ERK, DNMT1, ITGAL	up	Regulated methylation metabolism	Inhibited PBMC activation, corrected immune imbalance
		CD70	down		

## 8 Treatment strategies and clinical translation

### 8.1 MSC-engineered metabolic regulation enhancement

In the inflammatory microenvironment of SLE, the high expression of proinflammatory factors such as IFN-γ induces metabolic reprogramming in bone marrow-derived BMSCs, characterized by reduced glycolysis and enhanced oxidative phosphorylation. They also upregulate the expression of IDO and PD-L1, significantly enhancing their ability to suppress abnormal self-reactive T cell proliferation and reduce anti-double-stranded DNA antibody levels (27, 92). This MSC engineering regulation mediated by IFN-γ pre-treatment exhibits a direct causal relationship with the aforementioned metabolic reprogramming: The enhancement of oxidative phosphorylation not only provides sufficient energy for BMSCs to maintain their activity and synthesize immune regulatory molecules in the inflammatory microenvironment, but also, by regulating intracellular signaling pathways, further promotes the upregulation of IDO and PD-L1 - IDO can decompose tryptophan to produce kynurenine, inhibiting T cell proliferation and inducing the generation of regulatory T cells (Tregs), while PD-L1, by binding to the PD-1 on the surface of T cells, initiates the immune checkpoint pathway, blocking the activation and proliferation of abnormal T cells, and the two synergistically amplify the immunosuppressive effect, thereby more efficiently correcting the immune dysregulation state of SLE. Clinical studies have confirmed that IFN-γ-pretreated human clonal BMSCs exhibit significantly enhanced immunosuppressive functions compared to untreated controls, more effectively alleviating immune dysregulation in SLE patients (27). Similarly, in RA, metabolically reprogrammed BMSCs suppress abnormal T and B cell activation by regulating metabolic pathways. This reduces the production of proinflammatory factors (e.g. IL-6 and TNF-α) and autoantibodies (e.g. anti-citrullinated protein antibodies) (52, 82). Autologous bone marrow-derived BMSC transplantation has successfully alleviated the clinical symptoms of refractory RA in Iranian clinical studies, confirming its safety and efficacy (82).However, the clinical translation and standardization of such IFN-γ pre-treatment protocols still face numerous challenges: Firstly, the parameters such as the concentration of pre-treatment IFN-γ, the duration of action, and the culture conditions lack a unified standard. Different studies adopt significantly different protocols, resulting in insufficient repeatability and comparability of the immunosuppressive effect; Secondly, the heterogeneity of BMSCs among individuals is relatively high. Under the same pre-treatment conditions, the metabolic reprogramming efficiency and immune regulatory ability of BMSCs from different donors (especially autologous patients) vary, making it difficult to achieve precise regulation of personalized treatment; Thirdly, the survival time, metabolic stability, and long-term immune regulatory effect of BMSCs in the body after pre-treatment are still unclear, which may affect the persistence of the treatment. All these issues need to be gradually resolved through large-sample preclinical studies and standardized clinical trials.

## 9 Combination metabolic drugs

Combining MSC therapy with small-molecule drugs that modulate metabolism can produce synergistic effects, representing a highly promising therapeutic approach. Relevant metabolic drugs and their corresponding metabolic pathways are currently under investigation and have demonstrated therapeutic efficacy in immune-mediated inflammatory diseases such as RA. For example, the AMPK pathway regulates mitochondrial function and lipid metabolism. This influences the adipogenic differentiation of BMSCs and sustains the cellular energy supply and antioxidant capacity under hypoxic conditions. This enhances BMSC survival and immunomodulatory efficiency within inflammatory microenvironments (33). Metformin, an AMPK activator, can synergistically inhibit pro-inflammatory Th17 cell differentiation by reprogramming immune cell metabolism. As a classic AMPK activator, metformin mainly exerts a synergistic effect by directly regulating the metabolic state of BMSCs: It can directly activate the AMPK signaling pathway within BMSCs, further strengthening their oxidative phosphorylation metabolic phenotype, while inhibiting the abnormal activation of the glycolytic pathway, reducing the secretion of pro-inflammatory metabolites such as lactic acid, thereby enhancing the immunosuppressive activity of BMSCs; On the other hand, metformin can indirectly influence the function of BMSCs by altering the inflammatory microenvironment, that is, by inhibiting the glycolytic reprogramming of immune cells such as macrophages and T cells, reducing the release of pro-inflammatory factors (such as IL-17, TNF-α), providing a more suitable microenvironment for the survival and immune regulation of BMSCs in the body. The two mechanisms jointly inhibit the differentiation of pro-inflammatory Th17 cells, and this mechanism has been verified in animal models of multiple sclerosis (MS). This strategy has demonstrated promising therapeutic effects in multiple sclerosis (MS) animal models (93). It is worth noting that the safety of combining metabolic drugs with MSCs requires careful assessment. Although drugs such as metformin are widely used clinically, the combination with MSCs may alter the pharmacokinetics of the drugs or trigger unknown interactions. For instance, in patients with SLE or RA, special attention should be paid to whether the risks of drug-related gastrointestinal reactions, lactic acidosis (for metformin), or hypoglycemia (for 2-DG) increase due to the combined treatment. Preclinical studies need to systematically evaluate the acute and chronic toxicity of the combined strategy, its impact on liver and kidney functions, and the potential risk of over-immunosuppression leading to infections. Further research suggests that NLRP3 inflammasomes influence BMSC glucose metabolism by regulating Glut1-mediated metabolic reprogramming (31). Similarly, the glycolysis inhibitor 2-deoxyglucose (2-DG) reduces excessive proliferation and inflammatory responses in the synovial cells of patients with RA by inhibiting abnormally active glycolytic pathways, offering a novel approach to controlling joint lesions (94–97). 2-DG mainly exerts its effects through indirect means. It achieves a synergistic effect by targeting the glycolytic process of immune cells and diseased cells in the inflammatory microenvironment: It can specifically inhibit the glycolysis of RA synovial cells and pro-inflammatory macrophages, reduce ATP production within the cells and the secretion of pro-inflammatory factors, and alleviate local inflammatory responses. At the same time, the improvement of the inflammatory microenvironment can alleviate the abnormal induction of metabolic reprogramming of BMSCs, maintain the oxidative phosphorylation phenotype and the stability of immune regulatory functions of BMSCs, and thereby form a synergy with BMSCs treatment, enhancing the anti-inflammatory effect. Determining the optimal timing, dosage, and administration route of combined therapy is of utmost importance. From the perspective of disease stage, in the early or active phase of immune-mediated inflammatory diseases (such as SLE and RA), the inflammatory microenvironment has the most significant inhibitory effect on the functions of BMSCs. At this time, combining metabolic drugs may maximize the “empowerment” of MSCs. For patients with refractory or relapsing conditions, a combined strategy can be used as a salvage therapy. In terms of the administration plan, sequential treatment may be required: for example, using metabolic drugs (such as metformin) for short-term “pre-treatment” to improve the internal microenvironment, followed by MSC infusion; or administering low-dose maintenance drug treatment for a long time after MSC infusion to prolong the efficacy. The administration route needs to take into account both systemic and targeted aspects: intravenous infusion is suitable for systemic SLE, while local intra-articular injection may be more suitable for active RA arthritis. The dose exploration needs to balance efficacy and safety and should be individualized titration based on drug blood concentration, MSC survival indicators, and clinical response. Future efforts may further optimize the therapeutic efficacy of MSCs in immune-mediated inflammatory diseases such as lupus by combining them with relevant metabolic drugs. Furthermore, identifying the patient subgroups that may benefit the most from such combined strategies is crucial for the advancement of precision medicine. The potential benefit subgroups include: 1) refractory patients who do not respond adequately or are intolerant to traditional immunosuppressants (such as hormones, methotrexate); 2) immune-mediated inflammatory diseases patients with metabolic abnormalities (such as insulin resistance, mitochondrial dysfunction), whose MSCs may have metabolic defects; 3) patients with high disease activity and significantly elevated levels of inflammatory factors (such as IFN-γ, TNF-α), as their MSCs are more prone to functional exhaustion and require metabolic drugs for support; 4) patients with specific autoantibody positivity (such as high anti-dsDNA antibodies in SLE) or specific imbalance of immune cell subsets (such as an increased Th17/Treg ratio), whose pathological mechanisms may be more related to targeted metabolic pathways.

## 10 Targeted delivery to tissues

Extracellular vesicles (EVs), which are secreted by bone marrow-derived BMSCs, can modulate B-cell responses by transferring bioactive molecules, such as microRNAs (miRNAs) and IL-6 (53). The advantage of EVs lies in their ability to circumvent the risks of immune rejection associated with cell transplantation, while also being amenable to engineered modifications that enhance targeting specificity. These advantages are particularly prominent in the treatment of SLE. They can avoid the immune compatibility issues that may arise from BMSC transplantation, and at the same time, precisely deliver bioactive molecules to regulate the functions of immune cells. For example, in SLE models, BMSC-derived exosomes improve the inflammatory microenvironment by suppressing B-cell activation, and autoantibody production, and promoting the differentiation of IL-10-secreting regulatory B cells (53, 54). In RA, these exosomes also interfere with the secretion of proinflammatory factors by macrophages (e.g. TNF-α, IL-1β), promoting the polarization of M1 macrophages towards anti-inflammatory M2 cells and alleviating joint inflammation (83). BMSC-derived exosomes are currently being explored in hematopoietic stem cell transplantation as a novel ‘cell-free therapy’ approach for treating immune-mediated inflammatory diseases (53).However, in the context of SLE treatment, the EVs targeted delivery strategy still faces many unique challenges, which limit its clinical application. Firstly, the *in vivo* targeting accuracy is insufficient: SLE is a systemic immune-mediated inflammatory disease that affects multiple organ systems. EVs are easily captured by the reticuloendothelial system such as the liver and spleen, making it difficult to efficiently concentrate them in the main lesion sites of SLE such as the kidneys and skin, resulting in reduced therapeutic efficacy. Moreover, the complex inflammatory microenvironment of SLE can interfere with the stability of the surface-modified targets on EVs, further weakening the targeting accuracy. Secondly, the production scalability is limited: clinical treatment requires a large amount of highly homogeneous EVs, but the existing separation and purification techniques (such as ultracentrifugation) have low yields, high costs, and the EVs secreted by different donor BMSCs have heterogeneity in composition and activity, making it difficult to achieve standardized large-scale production, which does not match the large-scale requirements of SLE clinical treatment. Thirdly, the loading efficiency and stability of drugs are poor: EVs naturally carry limited bioactive molecules, and when exogenous anti-inflammatory drugs, miRNAs, etc. are loaded, loading efficiency is low and molecules leak out. Moreover, the oxidative stress microenvironment in SLE may damage the structural integrity of EVs, leading to premature release of loaded molecules and affecting the therapeutic effect. In addition, the pharmacokinetic characteristics, long-term safety, and immunogenicity of EVs in SLE patients (although lower than cell transplantation, there are still potential risks) also need to be further clarified through preclinical research. (**Table 3**).

**Table 3:** Clinical and translational studies of MSC/MSC-EV therapy in SLE.

Therapy type	Study design	Sample size	Key findings	Major limitations
BMSCs Infusion BMSCs Infusion	Phase I/II Clinical Trial (Single-arm)	Single-center, typically 10–30 patients	Reduced SLEDAI score & anti-dsDNA antibodies; improved renal function; increased Tregs.	Lack of control group; heterogeneous protocols; unclear long-term efficacy.
	Animal Model (e.g., MRL/lpr mice)	Typically 6–10 mice per group	Prolonged survival; attenuated nephritis; reduced autoantibodies.	Species differences; immunorejection in xenogeneic models.
Engineered BMSCs	Preclinical (*in vitro* / animal)	Cell culture or small-scale animal studies	IFN-γ pretreatment enhances IDO expression and immunomodulatory potency.	Lack of standardized protocol; unknown *in vivo* safety and donor variability.
MSC-EVs Therapy	Animal Model Studies	Typically 5–8 mice per group	Improved proteinuria & renal pathology; suppressed B-cell hyperactivation.	Complex manufacturing; poor tissue targeting; no clinical data available.
MSC-EVs Mechanism	*In Vitro* Cell Studies	Cells from patients or healthy donors	Inhibited B-cell differentiation & antibody secretion; modulated cytokines.	Cannot replicate the complexity of the *in vivo* microenvironment.

## 11 Conclusion

SLE is a complex immune-mediated inflammatory disease in which an imbalance in the bone marrow microenvironment is a key pathogenic factor. As core stromal cells within this environment, BMSCs exert immunoregulatory effects through metabolic reprogramming and are emerging as a novel therapeutic target for SLE. This review clarifies how proinflammatory signals in the SLE inflammatory microenvironment prompt BMSCs to transition from metabolic plasticity towards a state of oxidative phosphorylation-dominant metabolic imbalance. This metabolic reprogramming reshapes lipid and amino acid metabolism, enabling BMSCs to actively regulate immunity.

Based on this metabolic reprogramming, BMSCs construct an extensive immunoregulatory network. Through secreted factors and extracellular vesicles, BMSCs can precisely modulate B cell function, suppress neutrophil NETosis, promote M2 macrophage polarization, induce tolerogenic dendritic cells, and correct the Th17/Treg balance. This multi-targeted approach reshapes the inflammatory microenvironment. Corresponding therapeutic strategies, such as the use of engineered BMSCs, the combination of BMSCs with metabolic drugs and the delivery of extracellular vesicles, have demonstrated potential in preclinical and early clinical studies.

However, challenges remain. The specific molecular mechanisms of metabolic reprogramming are not fully elucidated, existing clinical evidence is limited and requires validation through large-scale trials, and the synergistic regulatory networks of BMSCs on different immune cells await elucidation through multi-omics technologies. To address the aforementioned bottlenecks, in the future, we should focus on the following four cutting-edge research directions to precisely break through the technical barriers: Firstly, apply spatial multi-omics technologies (combining spatial transcriptomics and spatial metabolomics) to analyze the spatially resolved metabolic interaction networks between BMSCs and immune cells in the bone marrow microenvironment of SLE patients, and clarify the precise sites and regulatory rules of metabolic substance exchange between cells; Secondly, utilize stable isotope-labeled metabolic tracing combined with *in vivo* imaging technology to dynamically track the temporal changes in the metabolic reprogramming of BMSCs in the body, and establish a quantitative correlation model between metabolic phenotypes and the activity of SLE disease; Thirdly, integrate genomic, transcriptomic, and metabolomic data using artificial intelligence and machine learning algorithms to construct a metabolic regulation network prediction model for BMSCs, and screen the optimal metabolic intervention targets and combined treatment regimens; Fourthly, develop SLE bone marrow organoids and humanized immune reconstitution mouse models to simulate the complexity of the human disease microenvironment, providing a preclinical efficient validation platform for novel therapies based on BMSC metabolic regulation. In conclusion, by focusing on metabolic reprogramming and combining engineering techniques to modify BMSCs and utilize their homing properties to reshape the bone marrow immune microenvironment, it is expected to provide a new approach for the treatment of bone marrow-related immune-mediated inflammatory diseases. In summary, deepening our understanding of metabolic reprogramming mechanisms in BMSCs is crucial for developing more effective, personalized SLE therapies.
